# Direct Synthesis of Carbon Nanotube Field Emitters on Metal Substrate for Open-Type X-ray Source in Medical Imaging

**DOI:** 10.3390/ma10080878

**Published:** 2017-07-29

**Authors:** Amar Prasad Gupta, Sangjun Park, Seung Jun Yeo, Jaeik Jung, Chonggil Cho, Sang Hyun Paik, Hunkuk Park, Young Chul Cho, Seung Hoon Kim, Ji Hoon Shin, Jeung Sun Ahn, Jehwang Ryu

**Affiliations:** 1 Department of Physics, Kyung Hee University, Seoul 02453, Korea; amargupta@khu.ac.kr (A.P.G.); sjpark1112@khu.ac.kr (S.P.); sjyeo@khu.ac.kr (S.J.Y.); 2 CAT Beam Tech Co., Ltd., Seoul Biohub, 117-3, Hoegi-ro, Dongdaemun-gu, Seoul 02455, Korea; jijung@catbeamtech.com (J.J.); cgcho@catbeamtech.com (C.C.); radpsh@gmail.com (S.H.P.); 3 Department of Biomedical Engineering and Healthcare Industry Research Institute, Kyung Hee University, Seoul 02453, Korea; sigmoidus@khu.ac.kr; 4 Department of Radiology, Asan Medical Center, University of Ulsan College of Medicine, Seoul 05505, Korea; cjsakura@naver.com (Y.C.C.); kimsh6713@gmail.com (S.H.K.); jhshin@amc.seoul.kr (J.H.S.)

**Keywords:** carbon nanotube, open-type X-ray system, field emission

## Abstract

We report the design, fabrication and characterization of a carbon nanotube enabled open-type X-ray system for medical imaging. We directly grew the carbon nanotubes used as electron emitter for electron gun on a non-polished raw metallic rectangular-rounded substrate with an area of 0.1377 cm^2^ through a plasma enhanced chemical vapor deposition system. The stable field emission properties with triode electrodes after electrical aging treatment showed an anode emission current of 0.63 mA at a gate field of 7.51 V/μm. The 4.5-inch cubic shape open type X-ray system was developed consisting of an X-ray aperture, a vacuum part, an anode high voltage part, and a field emission electron gun including three electrodes with focusing, gate and cathode electrodes. Using this system, we obtained high-resolution X-ray images accelerated at 42–70 kV voltage by digital switching control between emitter and ground electrode.

## 1. Introduction

Carbon nanotubes (CNTs) show brilliant properties that make them amazing material for various nanoelectronic device applications including electron emitters [1,2,3]. The high aspect ratio, high thermal conductivity, and low chemical reactivity characteristics of CNTs make them the best material for the X-ray source in radiographic applications [4,5]. X-ray sources with CNT emitters have several benefits over their thermionic counterpart, including rapid response, cold cathode and miniature system [6]. Moreover, tunneling of electrons from the tips of the nanotube due to high local electrical field makes CNTs an ideal material for a cold cathode [7]. Thus, the CNT based cathode has emerged as a new imaging technology, which is presently being used as a micro CT scanner in the medical field [6].

The X-ray system, depending on the cathode-anode assembly inside the tube, is divided mainly into open and sealed type. A sealed type X-ray system comprises a vacuum sealed X-ray tube, which is smaller in size and cheaper, has smaller resolution, magnification and relatively shorter life. The distance between cathode and anode is fixed and there is a limited X-ray cone angle. In contrast, in an open type X-ray system, the vacuum pump is attached to the system and the distance between cathode and anode is adjustable which makes the focal spot closer to the sample achieving higher resolution and magnification [8]. Moreover, in X-ray imaging, one of the prime objectives is to observe the internal condition and structure of samples at non-destructive, high resolution and magnified condition [9]. In addition, to obtain high quality X-ray images, a stable electron beam is required, which is only possible by creating ultra-high vacuum condition (10^−7^ to 10^−8^ Torr) inside the chamber containing electron gun. Therefore, to achieve these objectives open-type X-ray system is preferred over sealed or closed type [10].

It has been reported that to use CNT as an electron emitter, the field emission (FE) characteristic should be excellent [11]. Many articles have been published reporting the several ways to improve the FE properties [12,13]. Most of them reported that for excellent FE, the CNTs should be grown vertically aligned on a treated, polished and patterned surface [14]. However, the patterning and polishing of the surface is time consuming and not so economic, which will ultimately make it hard for commercializing the CNT based cathode and X-ray system and make it difficult to compete with conventional filament based system in market. Normally, to fabricate the CNTs through cost effective and simple ways, metal substrate is preferred because of native embedded catalysts such as Ni, Fe, Cr etc. and low resistivity. Moreover, the CNTs can be grown directly on metal substrate without following the processes like sputtering, polishing, etc. [15]. The CNTs grown on non-polished metal surface are generally non-aligned and spiral shaped [16]. Some papers have reported that the non-aligned CNT emitters can produce satisfactory FE [17,18].

In this paper, we report the development of a portable open-type X-ray system and electron gun based on CNT cathode directly grown on non-polished raw metal substrate (Hitachi metal corp., Tokyo, Japan, YEF 426, Ni-42%, Fe-52%, Cr-6%) that is able to work under an ultra-high vacuum condition producing sufficient FE to be used as an X-ray source. The cost effective and direct way of synthesizing CNTs on non-polished metal substrate through direct current-plasma enhanced chemical vapor deposition (DC-PECVD) has been discussed. The FE properties of grown CNT emitter have been characterized. Lastly, using this system, the X-ray images of a computer mouse at various accelerating anode voltages, printed circuit board and rat were obtained to characterize the quality and resolution of X-ray images.

## 2. Experimental Section

### 2.1. Synthesis of CNT on Non-Polished Metal Substrate

**Figure 1:**
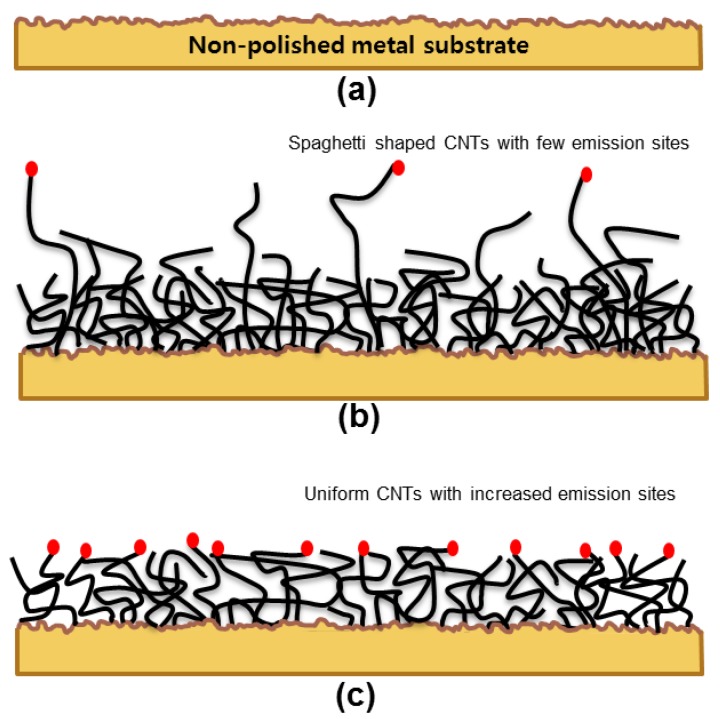
Process flow of (**a**,**b**) growth of carbon nanotubes (CNTs) emitter on metal substrate and (**c**) electrical aging treatment.

The CNTs were grown using a DC-PECVD explained in detail elsewhere [19]. For the PECVD process, rectangular rounded metal substrates with an area of 0.1377 cm^2^ and width of 1.5 mm, were used to grow the CNTs as in Figure 1a placed on a graphite susceptor. Before putting the metal substrates inside the PECVD chamber, the substrates were cleaned using acetone and isopropyl alcohol for 10 min each in an ultrasonic bath.

The PECVD process included three basic processes, namely pre-treatment, growth and post treatment. First, the necessary condition for PECVD process was obtained by creating the base pressure of 3.5 × 10^−7^ Torr and heating the susceptor to 900 °C. Secondly, in the pre-treatment process, the NH_3_ gas was inserted and pressure was maintained at 4.3 to 9 Torr for 10 min to form the nucleation sites on surface where CNTs can grow easily. Followed by growth process, C_2_H_2_ was inserted along with NH_3_ gas with the flow rates of 30 and 70 sccm, respectively. During this process, the susceptor voltage, the mesh voltage and the temperature was maintained at −300 V (0.011 A), 313 V (0.01 A) and 900 °C, respectively. The total pressure of the chamber was maintained at 4 to 6.5 Torr depending upon the shape of plasma above the graphite susceptor. The growing time was 20 min. Lastly, the post treatment was done for 1 min without biasing the electrodes. As shown in the Figure 1b, the spaghetti shaped CNTs were obtained.

### 2.2. Electron Microscopy

The morphology and quality of the CNTs were analyzed using field emission scanning electron microscope (SEM, Hitachi SU-70, Hitachi High-Technologies Corporation, Tokyo, Japan). The CNTs sample were sputtered-coated in Pt for 1 min before taking SEM images.

### 2.3. Field Emission Measurement of as-Grown CNT Emitter by Electrical Aging Treatment

**Figure 2:**
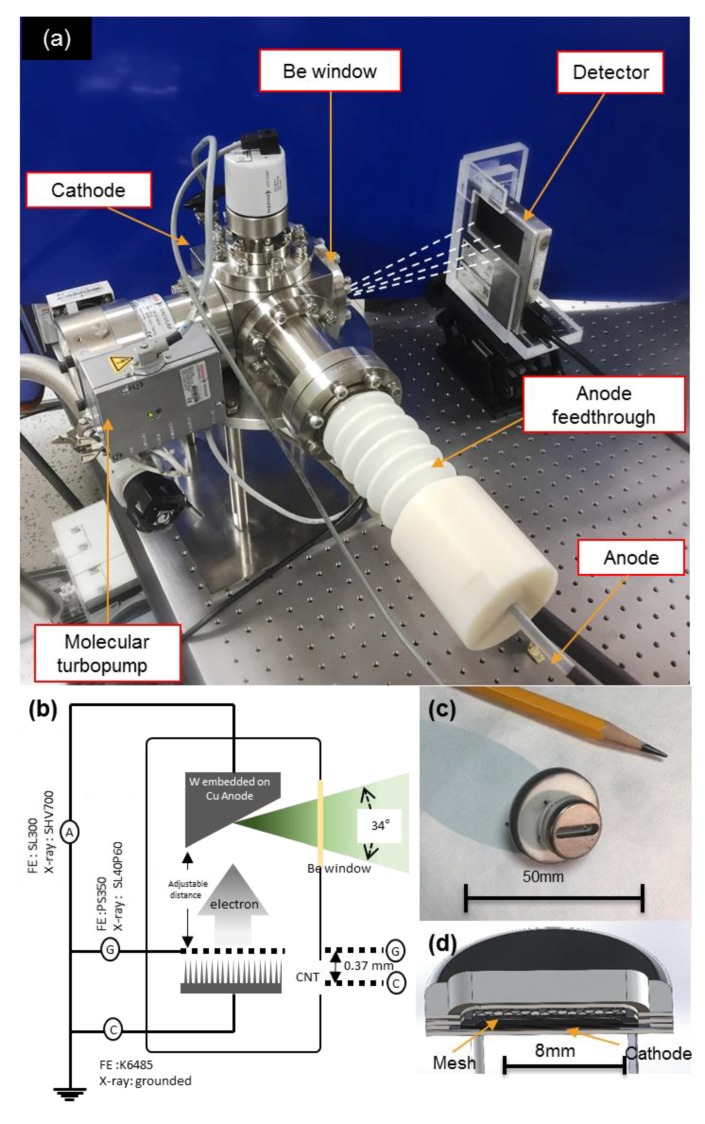
Photo image of (**a**) the open-type X-ray system and (**b**) schematic of triode configuration during FE test and inside the X-ray system (**c**) optical image of electron gun and (**c**) cross-sectional 3D design of electron gun.

Figure 1b shows the expected result of growth process. Generally, the CNTs grown on metal substrate has few electron-emitting sites because of the non-uniform heights of CNTs. In these conditions, the tip of taller CNT filaments only act as the emission site during FE [20]. Thus, to increase the emission sites and to enhance and stabilize the FE over a wide current range, the FE measurement is done by electrical aging treatment process [21]. The electrical aging treatment increases the emission sites by making the height of CNTs over a metal substrate uniform. We performed the repeatable FE measurement for 50 cycles to apply an electrical aging effect on the emitter. Figure 1c shows the expected result of electrical aging treatment. To measure the stable FE of CNT emitters, 50 more repeated cycles were performed. The FE properties of the CNT emitters were measured in the e-gun test chamber with triode configuration and base pressure maintained at 3.0 × 10^−7^ Torr. Figure 2b shows the triode configuration during field emission test. The triode configuration was preferred to avoid the module problems, such as anode melting and arcing effect between gate and cathode. Spellman SL300 (Spellman High Voltage Electronics Corporation, New York, NY, USA) was chosen to measure anode current and as an anode voltage source. Anode voltage of all field emission measurements were obtained below 8 kV. For gate voltage source and gate current measurement, the Stanford research PS350 (Stanford Research Systems, CA, USA) was used. For the cathode current measurement, Keithley 6485 (Keithley Instruments, Inc., OH, USA) was installed between the emitters and the ground. The gap between the mesh and the anode was maintained at 370 μm.

### 2.4. Description of the X-ray System

The X-ray system, shown in Figure 2a, is placed on and connected to a circular slab held by 4 rods connected to a rectangular slab, which in turn is mounted on an optical breadboard. It consists of a 4.5-inch cubic shaped vacuum chamber with 723.83 cm^3^ of internal volume. The faces of the cubic interface had the following sub-system: (i) a customized high voltage electrical feedthrough connected to the reflection anode, i.e., a 5.5 mm radius tungsten brazed onto a Cu anode with a diameter of 19 mm, sliced at 17° angle; (ii) a 4-pin electrical feedthrough connected to an electron gun and a vent line at the top of interface to fill the chamber with air; (iii) a beryllium window of thickness 0.254 mm for X-rays transmission; (iv) a molecular turbopump (TMU 071P, Pfeiffer Vacuum Technology, Berlin, Germany) connected to a rotary pump; (v) a full range gauge (PKR251, Pfeiffer Vacuum Technology, Berlin, Germany) to measure the pressure inside the chamber. The electron gun, shown in Figure 2b has a diameter of 16 mm and consists of focusing, gate and cathode electrodes. The cathode is connected to a rectangular rounded metal substrate with an area of 0.1377 cm^2^ and width of 1.5 mm. The gate mesh is a regular hexagon whose side is 360 µm, has an area of 0.00336 cm^2^ and aperture ratio of 89.7%. The focusing electrode has length of 10 mm, open width of 3 mm and thickness of 2.5 mm. The distance between gate and cathode is maintained at 370 μm. The three different electrodes of electron gun are connected to three different pins of cathode feedthrough. The cathode and the anode are installed opposite to each other, while the Be window and turbopump connections are at a right angle to both. The molecular turbopump is maintained at a base vacuum level of 10^−8^ Torr. The weight of the system is 20 kg without the turbopump weight. Figure 2b shows the schematic of triode configuration inside the X-ray system. Two power sources were used for X-rays generation, i.e., the anode voltage was supplied by SHV700 power supply (Convertech, Seoul, Korea) capable of delivering 1–70 kV, the gate voltage was supplied by Spellman High Voltage SL40P60/NSS/100 (Spellman High Voltage Electronics Corporation, New York, NY, USA) power supply capable of delivering 0–40 kV and cathode electrode was grounded.

## 3. Results and Discussion

### 3.1. CNT Emitters for Open Type X-ray Source

**Figure 3:**
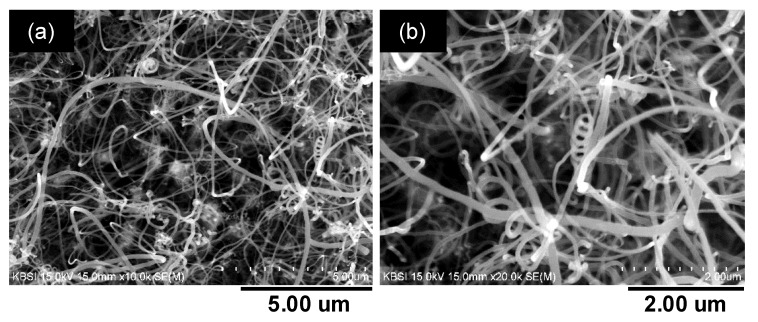
SEM images of the CNT forest at (**a**) 10.0 k and (**b**) 20.0 k magnification.

Figure 3 shows the SEM images taken at 5 μm and 2 μm scale, respectively. The CNTs are spiral and appear as spaghetti like structures grown on an ocean of catalysts such as Ni, Fe and Cr. The uniform distribution of CNTs on metal surface confirms the presence of adequate amount of suitable catalyst nickel needed for the formation of seeds. This result further confirms that the CNTs can be directly grown on non-polished and raw metal substrate easily, which reduces the thin film deposition processes like sputtering with Ni, treatment with acidic reagents and so on.

### 3.2. Field Emission Properties of CNT Emitters

**Figure 4:**
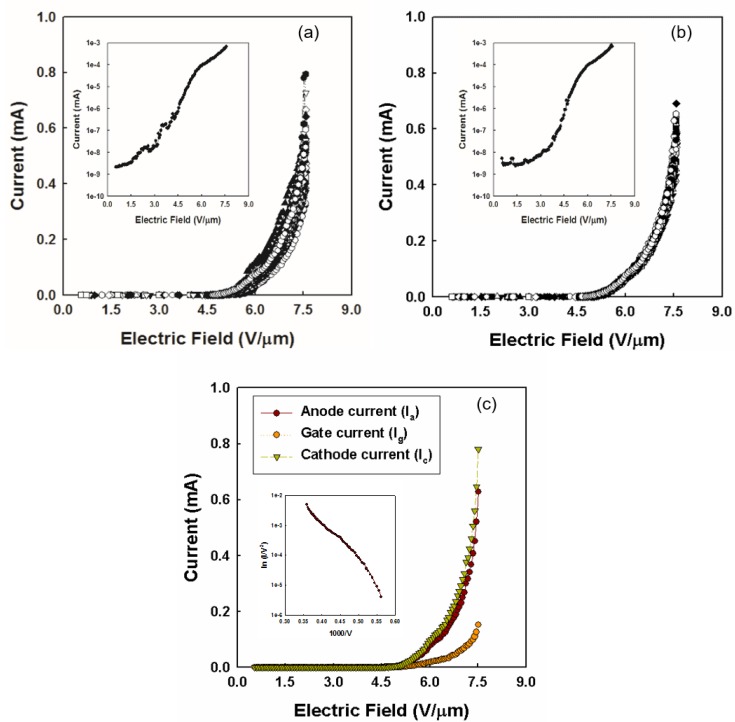
Field emission characteristic of CNT-emitter (**a**) during the electrical aging treatment; inset is the semi log plot of cathode current at 50th cycle (**b**) after the aging treatment; inset is the semi log plot of cathode current at 100th cycle and (**c**) at the last 100th cycle; inset is the Fowler Nordhiem (FN) plot.

After the electrical aging treatment, CNTs with uniform height and increased electron emission sites were obtained. The FE properties of CNT emitters are shown in Figure 4. Figure 4a shows the FE measured during the electrical aging treatment (1st–50th Cycles). As the tall CNT emitters on metal substrate can only act as electron emitters, with the electrical aging treatment, the average height of CNT emitters become uniform, which gradually increases the emission sites and stabilizes the FE. Figure 4b shows the stable FE after electrical aging treatment.

Table 1 shows the change in the stability of FE over the cycles at operational voltages and electric fields for X-ray imaging. This table further confirms the result of electrical aging treatment. During the 1st–50th Cycles, the standard deviation (S.D.) of anode current and relative standard deviation (R.S.D.), i.e., the percentage of ratio between S.D. and average current, were observed to be high. This data signifies that the FE during this cycle was unstable. However, during the 51st–100th Cycles, the S.D. and R.S.D decreased producing stabilized FE. For example, the S.D./R.S.D. from the 1st–50th Cycles to the 51st–100th Cycles at electric field of 7.6 V/µm decreased by almost 2 times. One thing that should not be forgotten is that there was no degradation of anode current over the cycles. This result also confirms the stability of anode current over the cycles. From the inset graphs of Figure 4a,b (i.e., the semi log plot FE of cathode current at 50th and 100th cycle, respectively) it can be observed that the turn-on electric field of CNT emitters was 4.5 V/µm. Figure 4c shows the FE graph at the 100th cycle and inset shows the FE phenomena by linearity of Fowler Nordhiem plot [22]. The cathode current of approximately 0.78 mA and anode current of 0.63 mA was obtained at a gate electric field of 7.51 V/µm. The anode current was measured by adjusting the gate electrode in the dc mode. The leakage current ratio was below 20%.

### 3.3. X-ray Images Using the 4.5-Inch Open-Type X-ray System

**Figure 5:**
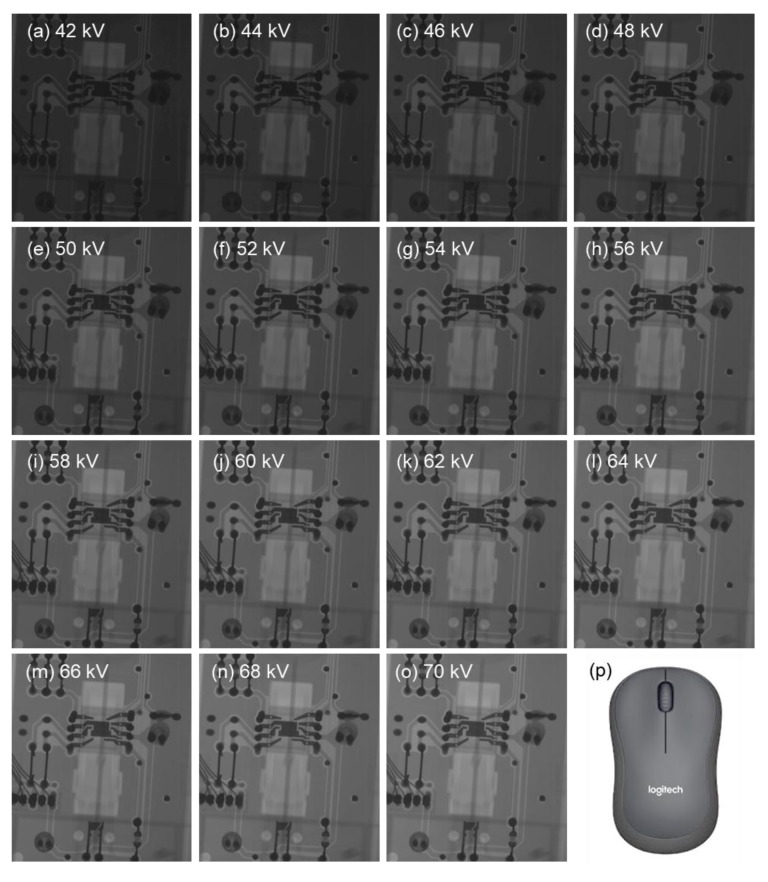
(**a**–**o**) X-ray images and (**p**) the optical image of computer mouse with accelerated energies 42–70 kV.

**Figure 6:**
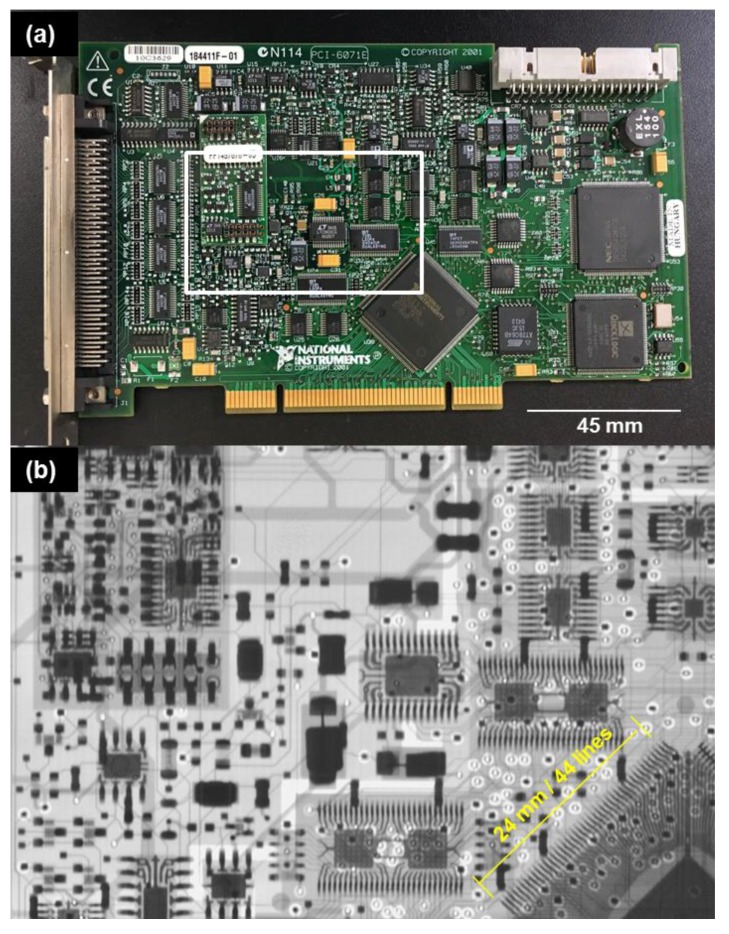
(**a**) Optical image and (**b**) X-ray image of printed circuit board taken at 45 kV/0.3 mA.

**Figure 7:**
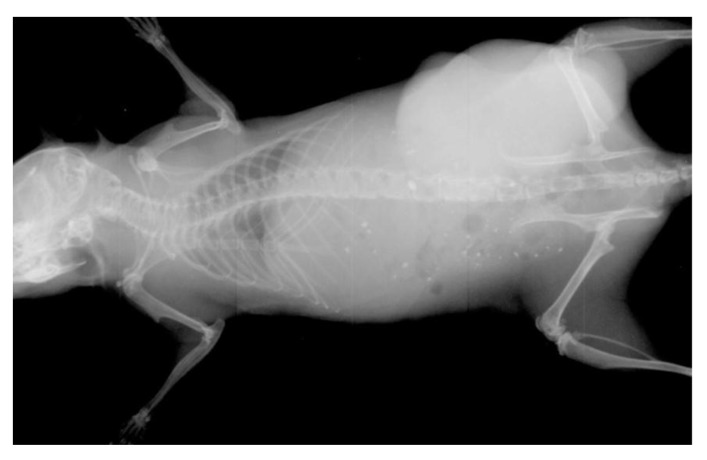
X-ray image of an alive rat taken at 55 kV/0.3 mA.

The thus developed open type X-ray system with CNT emitters was operated from 42 to 70 kV to take the X-ray images of computer mouse, printed circuit board and rat. Figure 5 shows the X-ray images of the computer mouse taken at different accelerating voltages. From these images, we found that the image with better brightness and contrast could be taken at bias voltage between 54–56 kV. To calculate the resolution of the X-ray image, the printed circuit board was exposed to 45 kV accelerated energy with 0.3 mA current for 5.0 s. Figure 6 shows the X-ray image of the printed circuit board and the resolution was found to be 0.2 mm. Figure 7 shows the X-ray image of the rat taken at 55 kV with an anode current of 0.3 mA exposed for 3.0 s. The detector (RAD icon, 0889, Teledyne Rad-icon Imaging Corp., CA, USA) with 1024 × 512 pixels was kept at a distance of 25 cm from the X-ray source to have better resolution of X-ray images.

## 4. Conclusions

The 4.5-inch portable open-type X-ray system and electron gun based on a CNT emitter has been demonstrated. The CNT emitters were grown directly on non-polished raw metal substrate using PECVD process produced sufficient FE and this has a promising result for X-ray generation. The metal substrate has made CNTs production simpler and more cost-effective. In addition to this, the field emission from CNT emitters grown on a metal surface can take X-ray images of animals and commercial products with high resolution and has the potential to be commercialized in the market in a medical field. Further research will be focused on the synthesis of CNT emitters on metal substrate by optimizing the parameters that can produce better FE.

## Figures and Tables

**Table 1:** Statistical analysis of field emission with cycles.

Voltage (V)	Electric Field (V/µm)	Cycle (1st–50th)			Cycle (51th–100th)		
		Average (µA)	S.D. * (µA)	R.S.D. * (%)	Average (µA)	S.D. * (µA)	R.S.D. * (%)
2000	5.4	17.61	5.10	28.94	19.06	2.57	13.48
2400	6.5	128.85	18.86	14.63	125.40	6.60	5.26
2800	7.6	511.46	93.15	18.21	562.26	48.98	8.71

* S.D.: Standard Deviation. * R.S.D.: Relative Standard Deviation.
